# Emerging Applications of Virtual Reality and Artificial Intelligence in Schema Therapy: A Scoping Review

**DOI:** 10.3390/healthcare14193292

**Published:** 2026-10-02

**Authors:** Flavia Salomoni Mansano, Karen Priscila Del Rio Szupszynski, Carmen Moret-Tatay, María José Beneyto-Arrojo, Margareth da Silva Oliveira

**Affiliations:** 1 Graduate Program in Psychology, Department of Psychology, Pontifical Catholic University of Rio Grande do Sul, Porto Alegre 90619-900, RS, Brazil; flaviasmansano@hotmail.com (F.S.M.); karen.delrio@pucrs.br (K.P.D.R.S.); marga@pucrs.br (M.d.S.O.); 2 Escuela de Doctorado, Universidad Católica de Valencia San Vicente Mártir, 46001 Valencia, Spain; mariajose.beneyto@ucv.es; 3 Faculty of Psychology, Catholic University of Valencia San Vicente Mártir, 46100 Valencia, Spain

**Keywords:** schema therapy, schema modes, early maladaptive schemas, digital tools, virtual reality, artificial intelligence, scoping review

## Abstract

Digital tools have become increasingly integrated into contemporary psychological practice, responding to the growing demand for accessible, scalable, and experiential mental health interventions. Within this evolving landscape, Schema Therapy represents a promising field for technological enhancement due to its emphasis on experiential techniques, mode work, and the restructuring of deeply rooted cognitive-emotional patterns. **Objectives:** This study aimed to map and characterize how Virtual Reality and Artificial Intelligence technologies are currently being used or proposed to support innovation in the application of Schema Therapy. **Methods:** A scoping review was conducted with searches performed across five major databases. Search strategies were adapted to each database, and records were screened according to inclusion/exclusion criteria. **Results:** Four records met eligibility criteria, revealing an emerging yet limited body of work dedicated to theoretical and experimental innovation in this area. Virtual Reality and metaverse-based interventions were proposed to create immersive, ecologically valid environments that facilitate emotional reprocessing, mode restructuring, and reductions in self-criticism, key mechanisms within ST. AI tools have demonstrated potential for large-scale identification of Early Maladaptive Schemas, enabling new forms of assessment and case conceptualization within online mental health contexts. **Conclusions:** No current evidence is sufficient to establish the efficacy of combining digital technologies such as Virtual Reality or Artificial Intelligence with Schema Therapy. The available literature remains limited and includes different stages of research development, from conceptual proposals and protocols to empirical studies. The identified applications primarily use these technologies as tools to support established Schema Therapy procedures rather than to modify its theoretical concepts or techniques. Although the findings indicate possible directions for technologically supported interventions, the current evidence is insufficient to establish their clinical effectiveness. Further empirical research is needed to evaluate their effects, feasibility, and clinical use within Schema Therapy.

## 1. Introduction

Schema Therapy (ST) is an evidence-based Psychotherapy that was originally developed for the treatment of personality disorders and has subsequently been applied to a broader range of psychological difficulties. A central feature of the model is the concept of Schema Modes, which refers to transient patterns of emotions, cognitions, and coping responses that emerge when underlying schemas are activated [1]. Schema Modes occupy an important position in both the theoretical formulation and clinical practice of ST because they provide a framework for understanding rapid changes in patients’ emotional and behavioral states and for guiding therapeutic interventions.

Despite the centrality of Schema Modes in ST, important questions regarding their activation and functioning remain unresolved. A literature review [2] on the experimental activation of Schema Modes identified only five published studies using procedures such as stress interviews, film clips, and art- and drama-based interventions. The authors highlighted substantial heterogeneity across procedures and samples, limited robustness and specificity of the induced effects, and discrepancies between therapist- and patient-reported activation. They concluded that validated and replicable experimental procedures and reliable measures of Schema Mode activation are still lacking and identified the development of appropriate experimental tools as a priority for advancing research on Schema Modes and empirically testing the theoretical assumptions of ST. These methodological challenges are relevant because experimental investigation of Schema Modes requires procedures capable of eliciting sufficiently meaningful cognitive and emotional experiences under controlled conditions. The literature review [2] describes how Schema Mode activation can be investigated through guided autobiographical recall, scripted imagery, and experimentally presented triggers, while emphasizing the need to carefully match the cognitive and emotional characteristics of the stimulus to the intended Schema Mode. This need for controlled yet experientially meaningful procedures creates a relevant conceptual basis for considering technological approaches capable of manipulating perceptual and situational features of therapeutic experiences.

Within this context, Virtual Reality (VR) has emerged as a technology with distinctive characteristics for psychological research and intervention. Unlike conventional digital interventions, VR can provide immersive, interactive, and controllable environments in which individuals can experience simulated situations from a first-person perspective. These properties have led to increasing investigation of VR across mental health conditions and psychological processes, including exposure, emotion regulation, social interaction, and imagery-based interventions [3,4]. The possibility of presenting standardized yet experientially rich scenarios may be particularly relevant to research questions requiring the controlled manipulation of emotional and situational stimuli. The potential relevance of VR to ST therefore extends beyond its use as a general therapeutic technology. Because Schema Modes are conceptualized as state-like configurations involving cognitive, emotional, and behavioral responses, technologies capable of creating controlled and immersive experiences may offer new possibilities for investigating how specific situations contribute to mode activation and how activation may subsequently influence the behavior. This is consistent with the research agenda proposed by the researchers in [2], who emphasize the need to identify the circumstances under which Schema Modes are activated and to examine their consequences for information processing and behavioral tendencies.

Alongside the development of immersive technologies, Artificial Intelligence (AI) has been regarded as a useful tool for psychologists and psychiatrists, as it can contribute to advances in mental health through the use of large-scale language models by predicting clinical improvement based on mathematical analyses [5,6]. However, it is necessary to recognize the limitations of AI in this field, as the systems are not sufficient on their own to provide psychological care or to replace human contact. This limitation stems from the inability to establish a genuine and deep connection with patients and to make a fully contextualized clinical judgment of symptoms, among other restrictions inherent to the technology [7]. However, these developments raise the possibility that AI could eventually support the VR operationalization of complex Psychotherapy constructs through adaptive dialogue, personalized content, or dynamic interaction, while also highlighting the need to establish how such technologies can be theoretically aligned with established psychotherapeutic models.

For Schema Therapy, this issue is particularly important because the application of emerging technologies cannot be understood solely in terms of whether VR or AI is effective in mental health care in general. It is necessary to determine whether and how these technologies can be aligned with the specific theoretical constructs, therapeutic techniques, and proposed mechanisms of change in ST. Considering the current research agenda on Schema Modes [2], which emphasizes the need for better tools to experimentally induce and assess modes and to investigate the conditions under which they are activated. However, the literature concerning emerging technologies and ST may encompass diverse technological approaches, therapeutic targets, methodological designs, and levels of empirical development. Consequently, a comprehensive characterization of this emerging field is needed before more focused questions concerning efficacy or mechanisms can be adequately addressed. Mapping the available evidence can clarify which ST techniques have been addressed through emerging technologies, how these technologies have been applied, and what types of evidence currently exist.

A scoping review is particularly appropriate for such an emerging and heterogeneous field because it allows understanding the characteristics and types of available evidence to be mapped across different study designs and sources, while also identifying areas in which further research is needed [8,9]. Accordingly, the present scoping review aims to map the available literature on the application of Virtual Reality and Artificial Intelligence within Schema Therapy, with particular attention to the technological approaches employed, the Schema Therapy components addressed, the populations and contexts investigated, the methodological characteristics of the studies, and the current state of the evidence.

Based on the gaps identified in the literature, the research question guiding this scoping review was: How have Virtual Reality and Artificial Intelligence been used or proposed for use with Schema Therapy? Accordingly, the review aimed to map the available evidence and characterize the ways in which these technologies have been applied or proposed within Schema Therapy.

## 2. Methods

This scoping review was conducted in accordance with the Joanna Briggs Institute (JBI) methodology for scoping reviews and reported following the PRISMA extension for Scoping Reviews (PRISMA-ScR) [10,11].

### 2.1. Eligibility Criteria

Records were included if they met the following criteria:

1. Technology: Records addressing the use or proposed use of Virtual Reality (VR), Augmented Reality (AR), Mixed Reality (MR), or Artificial Intelligence (AI) in relation to Schema Therapy.
2. Schema Therapy relevance: Records explicitly referring to Schema Therapy or to established Schema Therapy constructs, techniques, or assessment frameworks, such as Early Maladaptive Schemas, Schema Modes, chairwork, imagery-based techniques, the Young Schema Questionnaire, or the Schema Mode Inventory.
3. Study design: Empirical quantitative, qualitative, or mixed-methods studies, including clinical trials, case studies, exploratory studies, pilot studies, feasibility or usability studies, as well as registered study protocols and theoretical or conceptual papers addressing the integration of these technologies with Schema Therapy.
4. Context: Clinical, experimental, assessment, research, or other contexts in which VR or AI was applied or explicitly proposed in relation to Schema Therapy.
5. Time frame: Records published between 2016 and 2026.
6. Language: Publications in any language for which the full text was available.
7. Publication type: Peer-reviewed journal articles, preprints, registered study protocols, book chapters, dissertations, and theses with accessible full text.

Records were excluded if they did not explicitly address the relationship between the selected technologies and Schema Therapy, or if the full text was unavailable, including conference abstracts, editorials, and website materials.

The methodological breadth is consistent with the exploratory nature of scoping reviews, in accordance with the guidelines of the Joanna Briggs Institute (JBI) [10]. The inclusion and exclusion criteria were defined to ensure the relevance and conceptual consistency of the analyzed records, thereby ensuring that the final sample comprehensively represented the available evidence on the proposed use of RV/IA technologies in the practice of Schema Therapy. In addition, theoretical literature combining Virtual Reality (VR) or Artificial Intelligence (AI) with Schema Therapy was included, as its discussions were considered interesting and capable of enriching the discussion in relation to the objective of the search.

### 2.2. Databases

The literature search was conducted on 8 June 2026 across five electronic bibliographic resources: PubMed, PsycINFO, Embase, LILACS, and the Cochrane Library. These resources were selected to provide broad coverage of the interdisciplinary literature relevant to the review question.

### 2.3. Search Strategy

The development of the search strategy was supported by technical staff from the PUCRS university library. The data search strategy was structured based on consultation of the Descriptores em Ciências da Saúde/Medical Subject Headings (Decs/Mesh). After defining the appropriate descriptors, the search strategy was structured based on four conceptual dimensions around which the research question was formulated (Schema Therapy/Psychotherapy, Virtual Reality, Artificial Intelligence). Controlled subject headings (MeSH, Emtree, APA Thesaurus) and free-text terms were used, combined with Boolean operators AND/OR to maximize the sensitivity and specificity of the search. No filters for language or study type were initially applied, in accordance with the recommendations of the Joanna Briggs Institute (JBI) for scoping reviews.

The terms “Virtual Reality,” “Artificial Intelligence,” “Schema Therapy,” and “Psychotherapy,” along with their derivatives and translations, were used according to the database consulted. Based on the PCC approach described in Table 1, the search strategy was structured using Boolean operators, combining “Schema Therapy” with “Virtual Reality” and/or “Artificial Intelligence,” and with “Psychotherapy.”

For scoping reviews, particularly when following JBI guidance, the PCC framework (Population, Concept, Context) is generally considered the most appropriate framework. Accordingly, the search equation followed the structure (Table 1).

Accordingly, the research question was formulated as follows: “How have Virtual Reality and Artificial Intelligence been used or proposed for use with Schema Therapy?

The general search strategy combined terms related to Schema Therapy, the technologies of interest, and Psychotherapy using Boolean operators. The search structure was: (“Schema Therapy” AND (“Virtual Reality” OR “Artificial Intelligence”) AND “Psychotherapy”). The OR operator was applied exclusively between the technology-related terms, allowing the retrieval of records addressing either Virtual Reality or Artificial Intelligence, while Schema Therapy and Psychotherapy were required concepts through the AND operator. The strategy was adapted to the syntax and indexing terms of each database. The specific search strategy used for each database is presented in Table 2.

### 2.4. Results Selection Process

Based on the searches, the results were saved and transferred to the Rayyan—Intelligent Systematic Review platform, where they underwent screening and were assessed for eligibility.

The 51 records identified were entered into the platform and screened for duplicates, resulting in 47 records for screening. Two reviewers independently and sequentially evaluated the titles and abstracts, followed by full-text assessment of the identified publications and final selection of the records included in the review. A conflict-resolution procedure was established, consisting of discussion between the two reviewers and, if necessary, consultation with a third reviewer, with discussion and review of the eligibility criteria among the three reviewers.

### 2.5. Data Extraction

In this scoping review, data from the included records were extracted using a data extraction form previously developed and pilot-tested by the review team. This procedure follows the methodological recommendations proposed by [8] and subsequently refined in the Joanna Briggs Institute manual, which advises that the form be developed interactively and aligned with the review’s objectives.

Data extraction from the included documents was conducted independently by one reviewer. At this stage, one record was identified as not meeting the eligibility criteria for inclusion in the review results. This was reported to and discussed with the second reviewer, who agreed with the decision to exclude the report based on the eligibility criteria. The reason for exclusion is described in detail in the Section 3.

To ensure that all variables necessary to answer the research question are adequately captured, data extraction was collected regarding the general characteristics of the studies, including author(s), year of publication, country, study type, research objective, digital resource used, sample, study objective, and main findings.

## 3. Results

A diagram created using the PRISMA Diagram Generator specifies the number of records obtained through database searches and how many were included after applying the inclusion/exclusion criteria.

**Figure 1:**
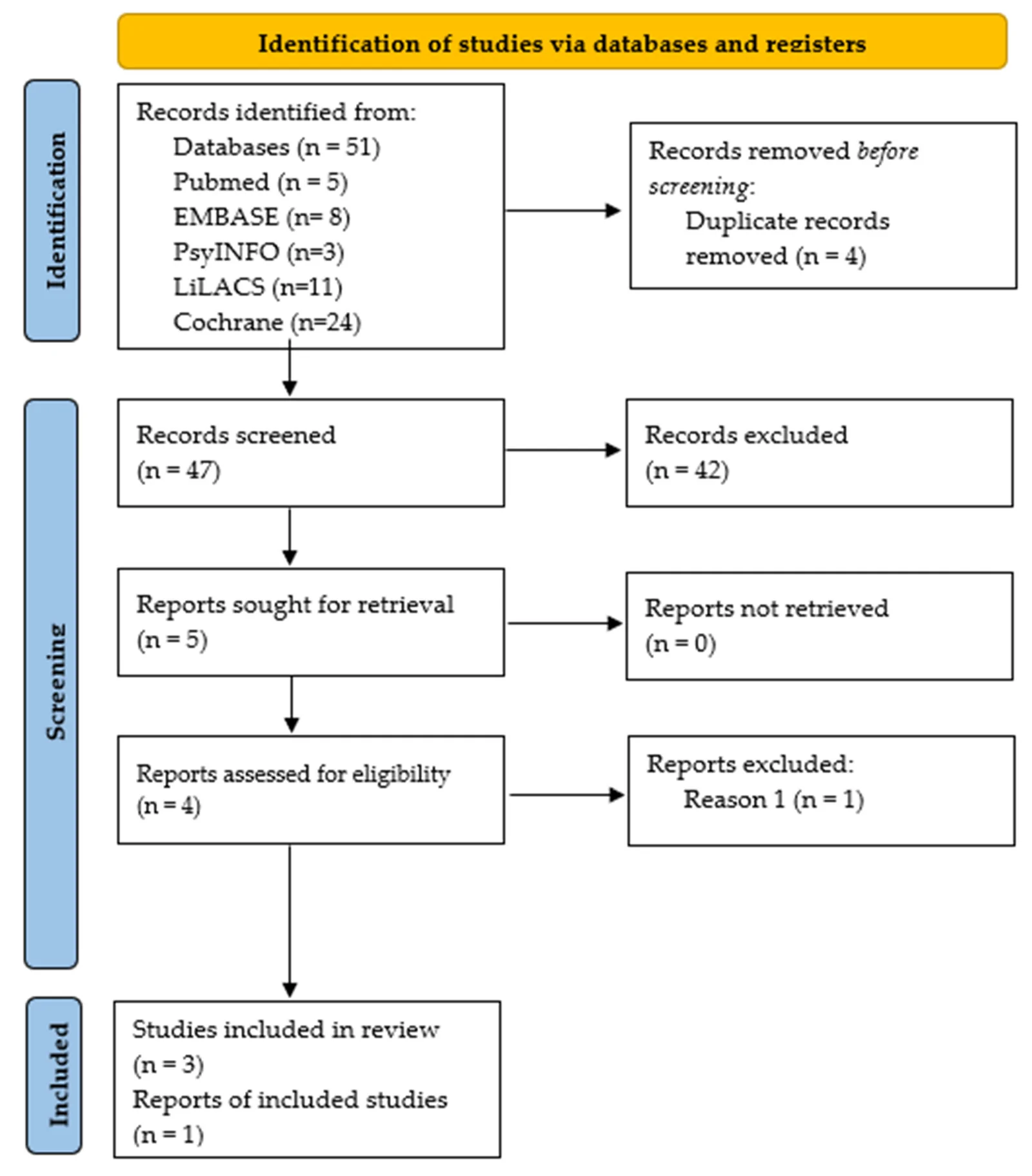
Study search flowchart—generated by the authors.

Figure 1 presents the flowchart of the process for identifying, screening, and including records in the review, according to the PRISMA model. Initially, 51 records were identified in different databases: PubMed (*n* = 5), EMBASE (*n* = 8), PsycINFO (*n* = 3), LiLacs (*n* = 11), and Cochrane (*n* = 24).

Prior to the screening stage, 4 duplicate records were automatically removed using the Rayyan tool, leaving 47 for initial evaluation. After reviewing the titles and abstracts, 42 records were excluded, resulting in 5 selected for full-text retrieval. All five reports were retrieved; one was excluded, and four were assessed for eligibility. The report was excluded because it did not meet eligibility criterion 1, which required studies to address the application of the technology within Schema Therapy. Full-text assessment indicated that the report proposed the use of Virtual Reality within Schema Therapy Coaching, an approach that differs from traditional Schema Therapy, which constitutes the scope of the present review. In addition, the proposed application was described only briefly and was not supported by references providing a theoretical or empirical basis for its use.

At the end of the selection process, four records were included in the review. A total of four records were included in the scoping review: one theoretical article, one experimental study, one empirical study, and one registered study protocol.

Following the identification and selection process presented in the PRISMA flow diagram, the included records were characterized according to their publication features. The characteristics considered were study, country, publication venue, type of publication, and journal evaluation based on JCR 2025 data released in June 2026. These publication characteristics are presented in Table 3.

The included records were further characterized according to their methodological and study-related features. The extracted information included study design, sample size, population, clinical condition, outcome measures, and main conclusions. These characteristics are presented in Table 4.

The following sections aim to systematically describe and organize the findings identified in the results. Critical analyses and interpretations of these findings are presented separately in the Section 4.

### 3.1. Digital Technologies Proposed

The use of Virtual Reality was explored more than the use of Artificial Intelligence, with two studies and one protocol proposing that VR could create immersive scenarios in which users interact with virtual characters representing Schema Modes.

In the clinical trial study [13], for example, participants interacted with an avatar representing their own self-critical voice, exploring the perspective-shifting process afforded by the experiential technique of chairwork to facilitate a dialogue between the internal critic, which the authors suggested could be understood as corresponding to two dysfunctional Parent Schema Modes, and the Healthy Adult Mode. Similarly, the study protocol proposes the use of Virtual Reality to conduct a mode dialogue based on chairwork in an interactive three-dimensional environment, with the aim of making these techniques more concrete and accessible for individuals who have difficulty imagining scenarios or characters during imagery rescripting exercises [14].

More specifically, in the theoretical article [15], Virtual Reality (VR) is presented as an early-stage technology that provides the technological foundation for the subsequent development of the metaverse. The authors theoretically discuss how broader VR environments, such as the metaverse, could provide a safe space for psychotherapeutic interventions and the reconstruction of emotional experiences that could be useful for working with Schema Modes.

The use of AI was identified in one study included in the review. The Large Language Models (LLMs), including the generative AI model GPT-4, were used to analyze large volumes of text from online mental health communities, seeking to identify associations between the 18 Early Maladaptive Schemas (EMSs) traditionally assessed by Young’s Schema Questionnaire and mental health problems [12].

The technologies identified for use or proposed use are described in the results presented in Table 5.

### 3.2. Types of Schema Therapy-Based Interventions

The identified Virtual Reality theorical proposal [15] and the experimental trial [13] were described in relation to their alignment with the fundamental principles of Schema Therapy. This description focused on whether the proposed technological applications preserved the conceptual and experiential foundations of the approach, particularly the use of Schema Modes as the central therapeutic framework.

In the experimental study [13], this technique was adapted for Virtual Reality through interaction with an avatar representing the “inner critic.” During the intervention, participants were instructed to respond assertively to the critical avatar, promoting the activation of the Healthy Adult Mode and reducing the influence of potentially dysfunctional modes, such as the Demanding Parent and Punitive Parent Modes. Similarly, the protocol proposal aims to compare chairwork performed using a traditional imagery exercise with its Virtual Reality version, investigating whether the visual and interactive representation of the modes may facilitate engagement and the effectiveness of the therapeutic exercise [14].

By proposing an AI-based analytical model grounded in Schema Therapy, the content analysis study [12] aimed to identify Early Maladaptive Schemas (EMSs) and cognitive and emotional patterns in posts from online mental health communities.

The Schema Therapy techniques that could potentially be facilitated by the technological applications proposed or used in each identified record are described in Table 6.

### 3.3. Samples Investigated

The clinical trial [13] was conducted with college students who exhibited high levels of self-criticism, identified through psychological screening instruments, with a total sample of 68 participants. The experimental protocol [14] proposes the inclusion of 30 participants aged 16 years or older who score high on specific Schema Modes, such as the Punitive Parent, Demanding Parent, or Vulnerable Child Modes, even in the absence of a formal clinical diagnosis.

The theoretical study proposing the use of VR in the metaverse [15] discusses the application of these technologies to clinical populations, particularly individuals with personality disorders, in whom Schema Modes play a central role in psychopathology; however, this application remains a theoretical proposal.

The AI study [12] analyzed 29,329 messages posted in online communities addressing mental health and psychological problems. Although the study did not provide specific sociodemographic information about the authors of the analyzed messages, the authors characterize the analyzed messages as a random sample and consider mental health problems to be prevalent among adults.

The sample or target sample investigated or proposed in each identified record is presented in Table 7.

### 3.4. Preliminary Efficacy Results

Among the results analyzed, only the randomized clinical trial provided empirical evidence regarding the effects of the intervention. The findings indicated that a single session of VR intervention produced significant immediate improvements in both groups, including reduced self-criticism (ηp^2^ = 0.190), increased self-compassion (ηp^2^ = 0.132), reduced negative affect (ηp^2^ = 0.119), and improved momentary self-compassion and self-esteem, with no significant change in positive affect [13]. Adding a third-person perspective did not result in greater improvements compared with the first-person condition, with both groups showing comparable gains. This finding suggests that responding assertively to the inner critic through interaction with the avatar may, in itself, promote immediate changes in these psychological indicators, with the authors suggesting a possible ceiling effect.

Exploratory analyses in the same study further showed significant associations between baseline Punitive Parent and Demanding Parent Modes and higher levels of self-criticism and lower levels of self-compassion. In contrast, the baseline Healthy Adult Mode was not significantly associated with either self-criticism or self-compassion. Participants in both conditions rated the intervention positively and reported that the exercise helped them gain perspective on their self-critical thoughts [13].

The exact *p*-values and between-group comparisons are described alongside the effect sizes for each outcome calculated by the RM-ANCOVA covariate model (Table 8) and correlations for the exploratory analysis (Table 9). However, confidence intervals are not reported by the authors in the published article, and for the exploratory correlations, the publication reports significance thresholds rather than exact *p*-values.

## 4. Discussion

The objective of this scoping review is to investigate how Virtual Reality and Artificial Intelligence are used or proposed for use in combination with Schema Therapy, aiming to map and identify how these technologies are developed and considered within the field. Across the records, both are proposed as facilitating tools for therapeutic interventions, rather than as modifications of Schema Therapy concepts or techniques, supporting the implementation of established therapeutic procedures.

The selected records indicate that these applications constitute a developing field of research, as reflected in the publication dates, the small number of records identified, and the methodological diversity of the included studies.

Following the methodological decisions adopted in the scoping review on positive schemas [16], protocols and conference papers may provide information that is insufficiently reliable or adequate for interpretation. Although the protocol was retained in the review and included in the Section 3, the information it provided was not considered in the Discussion. It is also noteworthy that the registered protocol was conducted by some of the same authors involved in the clinical trial. This decision was considered appropriate given that the objective of this scoping review was to map and understand how the proposed use of technologies such as VR and AI is being considered for combination with Schema Therapy. Accordingly, only information from studies published in indexed databases was considered in the Discussion (Table 10), with the necessary caution.

Since only the clinical trial [13] has an experimental design, it is the only identified study that relates to the perspective of regarding the use of experimental procedures to investigate the activation of emotional states associated with Schema Modes [2]. The authors emphasize the importance of experimental studies to investigate the circumstances under which Schema Modes are activated and the effects associated with such activation. They also note that experimentally induced mode activation is likely to be considerably shorter than mode activation occurring in real-life situations. Therefore, experimentally induced activation should not be considered equivalent to naturally occurring mode activation but rather as an opportunity to investigate the duration and characteristics of such activation [2].

However, the clinical trial [13] did not specifically assess changes in Schema Modes following the intervention but rather emotional states. Exploratory analyses conducted in the same study further showed significant associations between baseline Schema Modes and higher levels of self-criticism and lower levels of self-compassion. Although these findings do not provide direct evidence of changes in or activation of Schema Modes, they are relevant to the interpretation of the intervention because the study is theoretically grounded in Schema Therapy constructs and examines relationships between Schema Modes and psychological constructs addressed within the approach. This represents an important limitation when considering the findings in relation to changes in Schema Mode scores. The study also has other methodological limitations, including its single-session design and the absence of a condition without VR. Nevertheless, its findings are relevant to future research in the area, particularly because it is described by the authors as the first study to investigate a Schema Therapy exercise using VR, which is consistent with the limited evidence identified in this scoping review. Given the preliminary nature of this evidence, considering these findings as evidence of the efficacy of combining VR with Schema Therapy would be premature.

An important distinction should therefore be made between studies that aim to induce or assess emotional states and those that aim to induce or assess Schema Therapy constructs. Although Schema Modes involve emotional states when activated, Schema Therapy conceptualizes modes as broader states that also encompass characteristic behaviors and cognitive beliefs [1]. Accordingly, Schema Therapy has specific instruments for assessing Schema Modes, such as the Schema Mode Inventory (SMI), which considers emotional states as well as the behavioral and cognitive characteristics associated with each mode. Thus, changes in emotional states may reflect aspects of Schema Mode activation or change but cannot in themselves be interpreted as evidence of changes in specific Schema Modes when these modes are not directly assessed.

This may be one of the points to consider when understanding the limited number of studies identified in this review. Extensive reviews have examined the use of VR and AI in combination with mental health solutions [17], as well as VR interventions for emotional disorders [18]. These studies indicate that research has investigated how VR can support processes such as presence and personalization in relation to emotional states, but not specifically the effects of these interventions on Schema Modes. In this context, studies such as those in [19,20,21] are close to what could be considered the application of VR/AI to Schema Therapy, but they do not constitute direct applications of the approach because they do not use Schema Therapy-specific terminology or assessment instruments.

Similarly, Ref. [5] examined the use of large-scale language models to predict clinical improvement based on mathematical analyses. Study [6] also approaches the use of LLMs by considering their potential to predict treatment outcomes in patients with borderline personality disorder. However, because it does not use specific Schema Therapy concepts or instruments, it cannot be considered a direct application of the approach.

Likewise, the findings of the AI study [12], which used AI to identify Early Maladaptive Schemas and their associations with mental health problems in online communities, should be interpreted with caution. Although the authors reported that the extracted data reflected characteristics of EMSs and confirmed that the associations identified were consistent with findings from previous research, this remains the only study identified in the present scoping review that directly applies AI to Schema Therapy in this context. Therefore, while it provides evidence of the feasibility of using AI to investigate EMSs and their associations with mental health problems, it represents a first piece of evidence within the scope of this review and should not be interpreted as sufficient evidence to establish the validity or broader applicability of this approach.

Additionally, the identified records present diversity in study designs, which is consistent with previous observations that a substantial proportion of research involving VR and AI in other therapeutic approaches consists of pilot studies, experimental protocols, and clinical trials with relatively small samples [17,18]. The findings also align with Tabassum et al. in showing that studies involving these technologies are frequently conducted in highly developed countries. This pattern is also observed in the present review, including among the conceptual proposals, whose authors are affiliated with institutions in developed countries. This may be relevant when considering the resources required to develop specific programs and software for the implementation of these technologies, as such resources may represent a barrier to conducting research in settings with more limited technological investment.

Although the results, whether considered individually or collectively, do not provide sufficient evidence to support conclusions regarding technological innovations in Schema Therapy, the practical and ethical considerations discussed by the authors are relevant and may be considered in light of recommendations from research on Schema Therapy and on the use of technological tools, respectively. For example, the experimental study [13] followed the recommendation that emotional induction procedures should be brief, previously validated, and conducted in a manner that minimizes psychological distress. Notably, this study was conducted before the publication of the research agenda on experimental studies of Schema Modes, yet its methodological approach is consistent with this recommendation.

Risks associated with AI models should also be considered. Models that do not incorporate adequate transparency and accountability measures into algorithm development may result in insufficient or inappropriate treatment and the potential worsening of users’ health conditions due to inaccurate analyses of large datasets across demographic groups [22]. In this context, the lack of access to demographic information about the authors of the messages analyzed in the study included in this review [12] represents a relevant limitation. The involvement of multidisciplinary professionals in the development and evaluation of AI tools may help reduce these risks, alongside continuous assessment of AI systems to identify discriminatory outcomes. Data protection and privacy should also be considered, as well as the importance of maintaining human involvement when AI tools are used and achieving an appropriate balance between technological support and human decision-making. In addition, informing patients when AI tools are used in intervention planning allows them to make informed decisions regarding their care [22].

There is a consensus across the studies regarding the need for further research on the combination of these technologies with Schema Therapy. All records emphasize the need to protect patients’ privacy and data confidentiality. The clinical trial and the theoretical study also emphasize the importance of informed consent and minimizing emotional harm, ensuring that the simulation of difficult situations, such as confronting critical voices or revisiting family dynamics, is conducted safely to avoid retraumatization and promote well-being. Safety also involves researchers’ and practitioners’ knowledge of both the intervention and the specific digital tools, including consideration of appropriate limits related to cognitive overload and cybersickness, such as motion-related nausea and dizziness associated with VR. These considerations indicate that clinical use should not be implemented indiscriminately and requires prior training of practitioners in both Schema Therapy and the use of the specific digital tools.

Finally, although this review identified a limited number of records through the database searches and therefore does not allow robust conclusions to be drawn, it achieved its objective of identifying how VR and AI have been applied in Schema Therapy and the contexts in which their combination with the approach has been proposed, while also identifying the limitations and challenges associated with this emerging field.

## 5. Limitations

The main limitations identified relate to the small number of studies specifically addressing the combination of emerging technologies, such as Virtual Reality and Artificial Intelligence, with Schema Therapy. Although such technological tools have been incorporated into therapeutic approaches, their application and validation in the context of Schema Therapy remain limited, which may have restricted the scope of the evidence identified in this scoping review. The limited number of records retrieved may also be attributed, in part, to the highly specific nature of the search strategy, which focused on concepts directly related to Schema Therapy. Consequently, some relevant literature may be underrepresented, as certain studies might be indexed using terms associated with Cognitive Behavioral Therapy (CBT) [21] rather than terms specific to Schema Therapy. Furthermore, a 10-year publication date filter was applied to the search strategy. Therefore, earlier studies that might otherwise have met the eligibility criteria were not captured in the present review.

Beyond these limitations related to the search strategy, an additional consideration may be relevant to the development of research in this field, although it has not been identified in the literature on experimental research in Schema Therapy. Theoretically, the activation of Schema Modes depends on the individual’s coping style. This characteristic implies the need to personalize each type of intervention according to the individual’s mode activation patterns, which may represent a considerable challenge in terms of sample characteristics and intervention development costs.

Another factor that may have contributed to the limited number of records identified is that potentially relevant studies may not explicitly refer to Schema Therapy, despite investigating constructs that are closely related to its theoretical model, such as self-compassion and self-criticism. For example, studies [23,24] have investigated these constructs using Virtual Reality but did not employ Schema Therapy instruments for assessment or explicitly address Schema Therapy concepts such as the Vulnerable Child Mode and Punitive Parent Mode. Despite growing interest in digital technologies for mental health, recent reviews indicate the need for more clinical trials and longitudinal studies to assess the long-term effectiveness of these interventions, while also highlighting that high development costs may limit their widespread implementation, especially in resource-constrained settings [3]. Nevertheless, these studies contribute to understanding how VR can facilitate experiences with emotionally corrective qualities, which may be particularly relevant to the experiential and theoretical foundations of Schema Therapy.

Taken together, these limitations should be considered when interpreting the relatively small body of evidence identified in this review and the current state of research on the application of these emerging technologies in combination with Schema Therapy.

## 6. Conclusions

To the best of our knowledge, this is the first scoping review to systematically map the available literature on the use and proposed use of Virtual Reality and Artificial Intelligence in combination with Schema Therapy.

The results of this review indicate that the field of combining digital technologies, such as VR and AI, with Schema Therapy is still in the early stages of scientific development. The presence of clinical protocols in the recruitment phase and conceptual articles suggests that the proposed applications remain in the early stages of research, methodological development, and empirical validation. In addition, the identified studies propose methodological innovations, particularly alternative ways of assessing Early Maladaptive Schemas and implementing specific Schema Therapy techniques, with VR and AI serving as tools that may facilitate these processes. These applications do not propose reformulations of Schema Therapy concepts or techniques; rather, they explore alternative technological means of accessing and operationalizing existing components of the approach. Furthermore, the identified applications do not suggest that these technologies should be used autonomously or indiscriminately by patients. Instead, they are primarily conceived as supportive or adjunctive tools for the therapist, which may, in the future, be incorporated into clinical practice within a therapist-guided framework grounded in Schema Therapy principles.

The positive trends in outcomes observed in studies of emotional exposure using VR could suggest the potential relevance of this approach to future research in Schema Therapy. However, this review does not provide sufficient evidence to draw such a conclusion, particularly given the extremely limited number of studies identified. Rather, this possibility may be considered a hypothesis to be investigated in future research.

Regarding clinical practice, the findings of this review do not provide sufficient evidence to support the combined use of VR or AI with Schema Therapy. Any clinical application of these technologies in combination with Schema Therapy should therefore be considered uncertain in terms of the likelihood of favorable outcomes and approached with caution. For experimental research, the findings highlight methodological challenges and limitations that may arise when investigating such combinations. Nevertheless, these challenges may also represent opportunities for future research and provide directions for further investigation into the potential integration of VR and AI with Schema Therapy.

## Figures and Tables

**Table 1:** The PCC approach.

P	Unrestricted/not specified
C	Applications of Virtual Reality and Artificial Intelligence
C	Schema Therapy/Psychotherapy

Source: Prepared by the authors.

**Table 2:** The complete search strings for each database.

Database	Search Strategy
PUBMED	((“schema therapy”[MeSH Terms] OR “schema therapy”[Title/Abstract] OR “schema-focused therapy”[Title/Abstract] OR “schema mode”[Title/Abstract] OR “early maladaptive schema*”[Title/Abstract] OR “schema model”[Title/Abstract]) AND ((“virtual reality”[MeSH Terms] OR “virtual reality”[Title/Abstract] OR “immersive environment*”[Title/Abstract] OR “virtual environment*”[Title/Abstract] OR “augmented reality”[Title/Abstract] OR “mixed reality”[Title/Abstract] OR “XR”[Title/Abstract]) OR (“artificial intelligence”[MeSH Terms] OR “artificial intelligence”[Title/Abstract] OR “machine learning”[Title/Abstract] OR “chatbot*”[Title/Abstract] OR “conversational agent*”[Title/Abstract] OR “affective computing”[Title/Abstract] OR “natural language processing”[Title/Abstract]))) AND (“psychotherapy”[MeSH Terms] OR “psychotherapy”[Title/Abstract] OR “psychological intervention*”[Title/Abstract] OR “clinical practice”[Title/Abstract] OR “mental health”[Title/Abstract] OR “emotion regulation”[Title/Abstract])
EMBASE	(‘schema therapy’/exp OR ‘schema-focused therapy’:ab,ti OR ‘schema mode’:ab,ti OR ‘early maladaptive sche-ma*’:ab,ti OR ‘schema model’:ab,ti) AND (‘virtual reality’ OR ‘immersive environment*’:ab,ti OR ‘virtual en-vironment*’:ab,ti OR ‘augmented reality’:ab,ti OR ‘mixed reality’:ab,ti OR ‘xr’:ab,ti OR ‘artificial intelligence’/exp OR ‘machine learning’:ab,ti OR ‘chatbot*’:ab,ti OR ‘conversational agent*’:ab,ti OR ‘affective computing’:ab,ti OR ‘natural language processing’:ab,ti) AND (‘psychotherapy’/exp OR ‘clinical practice’:ab,ti OR ‘mental health’:ab,ti OR ‘emotion regulation’:ab,ti) AND [2016–2026]/py
PsycINFO	Index Terms: schema therapy AND Index Terms: virtual reality OR Index Terms: artificial intelligence AND Index Terms: psychotherapy
LiLACS	(schema therapy) AND ((virtual reality) OR (artificial intelligence)) AND (psychotherapy)
Cochrane	“schema therapy” in Title Abstract Keyword AND “virtual reality” in Title Abstract Keyword OR “artificial intelligence” in Title Abstract Keyword AND “psychotherapy” in Title Abstract Keyword–thesaurus descriptor

Source: Prepared by the authors.

**Table 3:** Publication characteristics of the included records.

Study, Year	Country	Publication Venue	Type of Publication	Journal Evaluation (JCR 2025 Data, Released June 2026)
Ang et al. (2025, JMIR) [12]	Singapore	Journal of Medical Internet Research (JMIR Publications)	Peer-reviewed empirical article	Impact Factor 8.2; Q1 in Medical Informatics (4/54) and in Health Care Sciences and Services (8/194).
Hidding et al. (2025) [13]	Netherlands	Journal of Behavior Therapy and Experimental Psychiatry (Elsevier)	Peer-reviewed empirical article	Impact Factor 2.3
VRAST (protocol, NCT07171736) [14]	Netherlands	ClinicalTrials.gov/Embase (trial registry record)	Registered clinical trial protocol (not a peer-reviewed publication)	Not applicable—trial registry record
Yin et al. (2022) [15]	China	Frontiers in Psychology (Frontiers Media)	Peer-reviewed Perspective article	Impact Factor 3.8; Q1 in Psychology (Multidisciplinary).

Source: Prepared by the authors.

**Table 4:** Methodological characterization.

Records	Study Design	Sample Size	Population	Clinical Condition	Outcome Measures	Main Conclusions
Ang et al. (2025, JMIR) [12]	Observational	29,329 posts	Anonymous online support seekers across 3 communities; no demographic data available	Anxiety, depression, PTSD, personality disorders, eating disorders, substance use disorders	Chi-square tests and odds ratios (EMS–condition associations); GPT-4-extracted EMS features across 5 case-conceptualization dimensions	Significant EMS–condition associations identified (e.g., anxiety–vulnerability to harm, OR 5.64; PTSD–mistrust, OR 5.04); depression, personality disorders, and PTSD linked to the broadest range of schemas
Hidding et al. (2025) [13]	Randomized controlled trial (single-session, parallel groups)	N = 68 (34 per group)	Dutch undergraduate psychology students	Excessive/subclinical self-criticism (non-clinical sample)	SCCS (primary); VAS; FSCRS; SMI; IPQ; study-specific evaluation questionnaire	Significant pre-post decrease in self-criticism/negative affect and increase in self-compassion; no added benefit of the perspective-change component; first study applying VR within a Schema Therapy exercise
VRAST (protocol,NCT07171736) [14]	Randomized crossover trial (protocol; not yet recruiting)	Planned N = 30 (not yet recruited)	Undergraduate students, ≥16 years, scoring >3 on Demanding Parent, Punitive Parent, and/or Vulnerable Child modes; healthy volunteers	Not applicable—healthy participants (non-clinical)	Unspecified “short assessment” pre/post each exercise version; post-session qualitative interview	Not applicable—protocol registered; not yet recruiting; no results available
Yin et al. (2022) [15]	Theoretical/Perspective article	Not applicable	Not applicable	Personality disorders	Not applicable—no data collected	Theoretical proposal only: argues the metaverse could give therapists a controllable “object world” to reshape maladaptive Schema Modes; no empirical support presented

Source: Prepared by the authors.

**Table 5:** Identified and proposed applications of Virtual Reality and Artificial Intelligence in Schema Therapy.

Reference	Tool Used/Suggested
Ang, Gollapalli, Du & Ng [12]	AI (LLM)
Hidding, Veling, Pijnenborg & Van der Stouwe [13]	VR
VRAST [14]	VR
Yin, Wang, Fei & Jiang [15]	Metaverse in VR suggested

Source: Prepared by the authors.

**Table 6:** Schema Therapy techniques supported by RV/IA.

Reference	Schema Therapy Technique Facilitated
Ang, Gollapalli, Du & Ng [12]	Assessment and identification of EMSs
Hidding, Veling, Pijnenborg & Van der Stouwe [13]	Mode dialogue
VRAST [14]	Mode dialogue
Yin, Wang, Fei & Jiang [15]	Assessment of Schema Modes, experiential exercises

Source: Prepared by the authors.

**Table 7:** Study samples and proposed populations.

Reference	Size	Sample/Proposed Population
Ang, Gollapalli, Du & Ng [12]	29,329 messages	Random sample
Hidding, Veling, Pijnenborg & Van der Stouwe [13]	N = 68	College students
VRAST [14]	Aimed N = 30	Participants aged ≥16 years with activated Schema Modes
Yin, Wang, Fei & Jiang [15]	Not applicable	Clinical populations

Source: Prepared by the authors.

**Table 8:** Clinical trial results.

Results	Outcome	Condition–Mean (SD)	F-Test Statistic Main Effect	*p*-Value	Effect Size
Primary outcomes	Self-criticism	1 PP: Pre 63.26 (12.44); Post 46.71 (14.28). 1 PP + 3 PP: Pre 67.15 (11.83); Post 52.97 (13.18)	Time: F(1, 65) = 15.26	<0.001 ***	ηp^2^ = 0.190
			Condition: F(1, 65) = 1.98	0.483	ηp^2^ = 0.030
			Time × condition: F(1, 65) = 0.50	0.483	ηp^2^ = 0.008
	Self-compassion	1 PP: Pre 47.00 (11.79); Post 60.32 (14.66). 1 PP + 3 PP: Pre 49.35 (14.66); Post 61.53 (18.37)	Time: F(1, 65) = 9.89	0.003 **	ηp^2^ = 0.132
			Condition: F(1, 65) = 0.58	0.451	ηp^2^ = 0.009
			Time × condition: F(1, 65) = 0.11	0.747	ηp^2^ = 0.002
Secondary outcomes	Positive affect	1 PP: Pre 66.29 (11.94); Post 67.57 (12.45). 1 PP + 3 PP: Pre 59.96 (18.66); Post 65.87 (15.87)	Time: F(1,65) = 2.69	0.106	ηp^2^ = 0.040
			Condition: F(1, 65) = 0.37	0.547	ηp^2^ = 0.006
			Time × condition: F(1, 65) = 0.84	0.364	ηp^2^ = 0.013
	Negative affect	1 PP: Pre 28.30 (17.57); Post 24.74 (15.93). 1 PP + 3 PP: Pre 26.74 (16.12); Post 21.97 (15.53)	Time: F(1, 65) = 8.77	0.004 **	ηp^2^ = 0.119
			Condition: F(1, 65) = 1.40	0.241	ηp^2^ = 0.021
			Time × condition: F(1, 65) = 0.06	0.810	ηp^2^ = 0.001
	Self-compassion and self-esteem	1 PP: Pre 60.60 (17.52); Post 70.88 (14.46). 1 PP + 3 PP: Pre 55.40 (17.14); Post 68.82 (14.16)	Time: F(1, 65) = 5.64	0.021 *	ηp^2^ = 0.080
			Condition: F(1, 65) = 0.32	0.576	ηp^2^ = 0.005
			Time × condition: F(1, 65) = 1.01	0.319	ηp^2^ = 0.015

Note: * *p* < 0.05, ** *p* < 0.01, *** *p* < 0.001 defined by Hidding et al. [13]. Source: Prepared by the authors.

**Table 9:** Clinical trial exploratory results.

Results	Modes	Total Sample Scores (SMI)	Correlations	
Exploratory analysis	Punitive Parent	M = 2.43 (SD = 0.73)	Baseline self-criticism	0.398 **
			Baseline self-compassion	−0.267 *
			Inadequate Self (FSCRS Subscale)	0.496 **
			Hated Self	0.612 **
			(FSCRS Subscale)	
			Reassured Self	−0.498 **
			(FSCRS Subscale)	
	Demanding Parent	M = 3.95 (SD = 0.75)	Baseline self-criticism	0.465 **
			Baseline self-compassion	−0.241 *
			Inadequate Self (FSCRS Subscale)	0.286 *
			Hated Self	0.231
			(FSCRS Subscale)	
			Reassured Self (FSCRS Subscale)	−0.319 **
	Healthy Adult	M = 4.20 (SD = 0.61)	Baseline self-criticism	−0.193
			Baseline self-compassion	0.205
			Inadequate Self (FSCRS Subscale)	−0.350 **
			Hated Self	−0.600 **
			(FSCRS Subscale)	
			Reassured Self (FSCRS Subscale)	0.567 **

Note: * correlation is significant at the *p* < 0.05 (2-tailed); ** correlation is significant at *p* < 0.01 (2-tailed) defined by Hidding et al. [13]. Source: Prepared by the authors.

**Table 10:** Articles considered in the Discussion of this Scoping Review.

Reference	Type
Ang, Gollapalli, Du & Ng [12]	Empirical study
Hidding, Veling, Pijnenborg & Van der Stouwe [13]	Randomized Clinica Trial
Yin, Wang, Fei & Jiang [15]	Theorical perspective

Source: Prepared by the authors.

## Data Availability

No new data were created or analyzed in this study. Data sharing is not applicable to this article.

## Abbreviations

The following abbreviations are used in this manuscript:

AI	Artificial Intelligence
EMS	Early Maladaptive Schemas
FSCRS	Forms of Self-Criticizing/Attacking and Self-Reassuring Scale
IPQ	Igroup Presence Questionnaire
SCCS	Self-Compassion and Self-Criticism Scale
SMI	Schema Modes Inventory
UMCG	University Medical Center Groningen
VAS	Visual Analog Scale
VR	Virtual Reality
VRAST	Virtual Reality-Assisted Schema Therapy

## References

[1] Young J.E., Klosko J.S., Weishaar M.E. (2003). Schema Therapy: A Practitioner’s Guide.

[2] Krans J., Hilgefort S., Lobbestael J. (2026). Experimental research on schema modes: Guidelines and research agenda. Curr. Psychiatry Rep..

[3] Emmelkamp P.M.G., Meyerbröker K. (2021). Virtual Reality Therapy in Mental Health. Annu. Rev. Clin. Psychol..

[4] Bell I.H., Pot-Kolder R., Rizzo A., Rus-Calafell M., Cardi V., Cella M., Ward T., Riches S., Reinoso M., Thompson A. (2024). Advances in the use of virtual reality to treat mental health conditions. Nat. Rev. Psychol..

[5] Guo Z., Lai A., Thygesen J.H., Farrington J., Keen T., Li K. (2024). Large Language Models for Mental Health Applications: A Systematic Review (Preprint). JMIR Ment. Health.

[6] Wibbelink C.J.M., Sellbom M., Grasman R.P.P.P., Arntz A., Sinnaeve R., Kamphuis J.H. (2025). Using the Minnesota Multiphasic Personality Inventory–2 Restructured Form to Predict Functioning after Treatment for Borderline Personality Disorder: A Machine Learning Approach. Psychol. Assess..

[7] Zhang Z., Wang J. (2024). Can AI Replace Psychotherapists? Exploring the Future of Mental Health Care. Front. Psychiatry.

[8] Peters M.D.J., Godfrey C., McInerney P., Khalil H., Larsen P., Marnie C., Pollock D., Tricco A.C., Munn Z. (2022). Best Practice Guidance and Reporting Items for the Development of Scoping Review Protocols. JBI Evid. Synth..

[9] Tricco A.C., Lillie E., Zarin W., O’Brien K.K., Colquhoun H., Levac D., Moher D., Peters M.D.J., Horsley T., Weeks L. (2018). PRISMA Extension for Scoping Reviews (PRISMA-ScR): Checklist and Explanation. Ann. Intern. Med..

[10] Aromataris E., Lockwood C., Porritt K., Pilla B., Jordan Z. (2024). JBI Manual for Evidence Synthesis.

[11] Arksey H., O’Malley L. (2005). Scoping Studies: Towards a Methodological Framework. Int. J. Soc. Res. Methodol..

[12] Ang B.H., Gollapalli S.D., Du M., Ng S.K. (2025). Unraveling Online Mental Health through the Lens of Early Maladaptive Schemas: AI-Enabled Content Analysis of Online Mental Health Communities. J. Med. Internet Res..

[13] Hidding M., Veling W., Pijnenborg G.H.M., van der Stouwe E.C.D. (2025). Facing Your Inner Critic: A Randomized Controlled Trial Investigating a Virtual Reality Intervention with and without a Perspective Change for Excessive Self-Criticism. J. Behav. Ther. Exp. Psychiatry.

[14] Clinical Trial Register (2025). VRAST: Virtual Reality Assisted Schema Therapy.

[15] Yin B., Wang Y.-X., Fei C.-Y., Jiang K. (2022). Metaverse as a Possible Tool for Reshaping Schema Modes in Treating Personality Disorders. Front. Psychol..

[16] van Donzel L., Claassen A.-M., Ouwens M.A., Broersen J., van Alphen S.P.J., Louis J.P., Videler A.C. (2026). Positive constructs in schema therapy: A scoping review. J. Clin. Psychol..

[17] Tabassum A., Ghaznavi I., Abd-Alrazaq A., Qadir J. (2025). Exploring the Application of AI and Extended Reality Technologies in Metaverse-Driven Mental Health Solutions: Scoping Review. J. Med. Internet Res..

[18] Rowland D.P., Casey L.M., Ganapathy A., Cassimatis M., Clough B.A. (2021). A Decade in Review: A Systematic Review of Virtual Reality Interventions for Emotional Disorders. Psychosoc. Interv..

[19] Sairanen E., Wallsten D. (2025). Facing Yourself in a Virtual Reality-Assisted Perspective-Taking Intervention: An Interpretative Phenomenological Analysis. Front. Psychol..

[20] Haber Y., Hadar Shoval D., Levkovich I., Yinon D., Gigi K., Pen O., Angert T., Elyoseph Z. (2025). The Externalization of Internal Experiences in Psychotherapy through Generative Artificial Intelligence: A Theoretical, Clinical, and Ethical Analysis. Front. Digit. Health.

[21] Van Kuik S., Van Emmerik A.A.P., Brinkman W.P., Uduwa-Vidanalage E., Schouten C., Arntz A. (2023). Efficacy and Acceptability of Virtual Reality Imagery Rescripting for PTSD due to Childhood Sexual Abuse: A Multiple Baseline Study. J. Evid.-Based Psychother..

[22] Olawade D.B., Wada O.Z., Odetayo A., David-Olawade A.C., Asaolu F., Eberhardt J. (2024). Enhancing mental health with artificial intelligence: Current trends and future prospects. J. Med. Surg. Public Health.

[23] Falconer C.J., Slater M., Rovira A., King J.A., Gilbert P., Antley A., Brewin C.R. (2014). Embodying Compassion: A Virtual Reality Paradigm for Overcoming Excessive Self-Criticism. PLoS ONE.

[24] Falconer C.J., Rovira A., King J.A., Gilbert P., Antley A., Fearon P., Ralph N., Slater M., Brewin C.R. (2016). Embodying Self-Compassion within Virtual Reality and Its Effects on Patients with Depression. BJPsych Open.

